# The Genome Sequence of *Rickettsia felis* Identifies the First Putative Conjugative Plasmid in an Obligate Intracellular Parasite

**DOI:** 10.1371/journal.pbio.0030248

**Published:** 2005-07-05

**Authors:** Hiroyuki Ogata, Patricia Renesto, Stéphane Audic, Catherine Robert, Guillaume Blanc, Pierre-Edouard Fournier, Hugues Parinello, Jean-Michel Claverie, Didier Raoult

**Affiliations:** **1** Structural and Genomic Information Laboratory, UPR 2589, IBSM, CNRS, Marseille Cedex, France,; **2** Unité des Rickettsies, UMR 6020, IFR 48, CNRS, Faculté de Médecine, Marseille Cedex, France; University of ArizonaUnited States of America

## Abstract

We sequenced the genome of *Rickettsia felis*, a flea-associated obligate intracellular α-proteobacterium causing spotted fever in humans. Besides a circular chromosome of 1,485,148 bp, *R. felis* exhibits the first putative conjugative plasmid identified among obligate intracellular bacteria. This plasmid is found in a short (39,263 bp) and a long (62,829 bp) form. *R.* *felis* contrasts with previously sequenced *Rickettsia* in terms of many other features, including a number of transposases, several chromosomal toxin–antitoxin genes, many more *spoT* genes, and a very large number of ankyrin- and tetratricopeptide-motif-containing genes. Host-invasion-related genes for patatin and RickA were found. Several phenotypes predicted from genome analysis were experimentally tested: conjugative pili and mating were observed, as well as β-lactamase activity, actin-polymerization-driven mobility, and hemolytic properties. Our study demonstrates that complete genome sequencing is the fastest approach to reveal phenotypic characters of recently cultured obligate intracellular bacteria.

## Abstract 2

*Rickettsia felis* is an obligate intracellular bacterium that lives in fleas and causes spotted fever in humans. Its genome sequence provides the first evidence that such bacteria can undergo conjugation.

## Introduction

Rickettsiae are obligate intracellular small gram-negative bacteria associated with different arthropod hosts. Many *Rickettsia* species infect human beings and are responsible for mild to severe diseases. *Rickettsia felis*, the agent of the flea-borne spotted fever rickettsiosis, exhibits several specificities among the currently recognized *Rickettsia* species. After being identified in fleas in 1990 [1], *R. felis* has been found worldwide in flea species such as *Ctenocephalides felis*, parasitizing cats and dogs, and *Pulex irritans*. *R. felis* is transovarially transmitted in these insects [2]. Several cases of human infection caused by *R. felis* have been reported [3,4]. *Rickettsia* species are phylogenetically classified into two groups: the typhus group and the spotted-fever group (SFG). *R. felis* belongs to the SFG, together with tick-associated *Rickettsia* species such as *R. conorii*, *R. sibirica*, and *R. rickettsii*. However its lifestyle resembles that of *R. typhi* (typhus group), which is also hosted and transovarially transmitted by fleas. Furthermore, *R. felis* is known to coinfect fleas with *Bartonella henselae*, *B. quintana*, and *Wolbachia pipientis* [5]. The culture conditions of *R. felis* were established in 2001 using *Xenopus laevis* tissue culture (XTC) cells at relatively low temperatures (optimally at 28 °C) [3]. Besides these features, little is known about this pathogen. To date, six *Rickettsia* genome sequences are available. These are from two typhus group species (*R. prowazekii* [6] and *R. typhi* [7]) and four SFG species *(R. conorii* [8], *R. sibirica* [9], *R. rickettsii*, and *R. akari)*. To further identify the specificities of *R. felis*, we determined its genome sequence.

## Results

### General Genome Features

**Figure 1:**
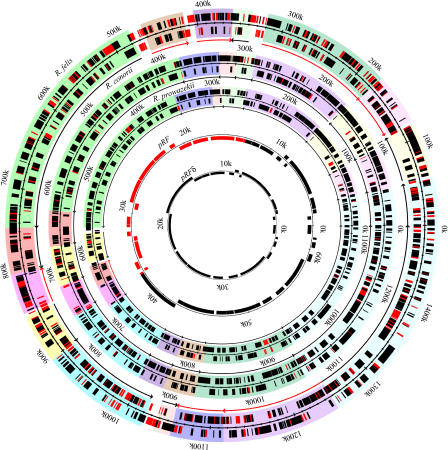
Circular Representation of *R. felis*, *R. conorii*, and *R. prowazekii* Genomes The three outer circles represent the chromosomes of *R. felis, R. conorii*, and *R. prowazekii*, respectively, with specific ORFs colored in red and nonspecific ORFs colored in black. Colinear genome fragments are highlighted by a shared background color, with their relative orientations indicated by arrows. The two inner circles represent two *R. felis* plasmids (pRF and pRFδ), with ORFs in the region unique to pRF colored in red.

The genome of *R. felis* comprises three replicons: a 1,485,148 bp circular chromosome and two circular plasmids identified for the first time in the genus *Rickettsia* (Figure 1). The predicted total complement of 1,512 protein-coding genes (open reading frames [ORFs]) is the largest among currently sequenced *Rickettsia* genomes (Table 1). Of these, 1,402 (92.7%) exhibited homologs in the nonredundant database and 1,080 (71.4%) were assigned putative functions.

**Table 1:**
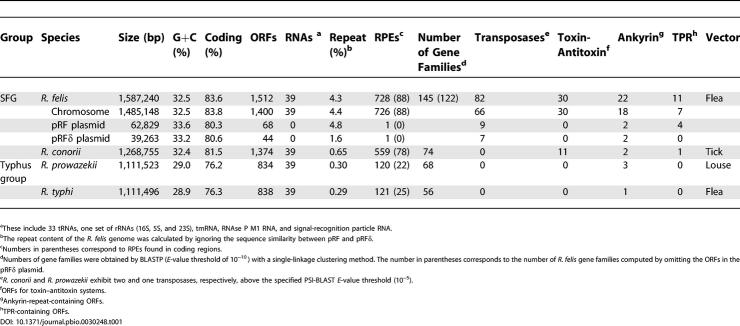
Comparison of *R. felis* and Other Published *Rickettsia* Genomes

**Figure 2:**
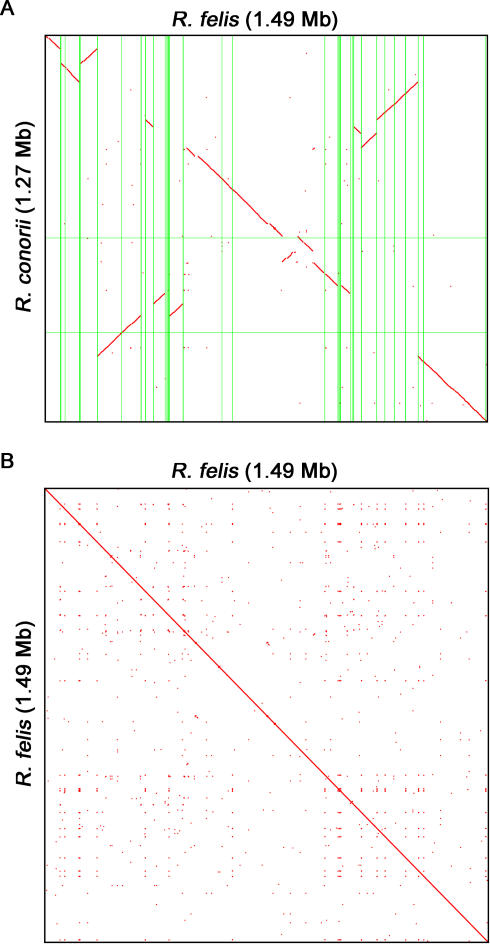
Genome Comparisons of *R. felis* and *R. conorii* Red dots represent homologous genomic segments greater than 150 bp identified by BLASTN (*E*-value < 10^−3^). (A) Comparison between *R. felis* and *R. conorii* chromosomes. Vertical and horizontal green lines indicate the positions of transposase ORFs in *R. felis* and in *R. conorii*, respectively. (B) Self-comparison of *R. felis* chromosome.

The *R. felis* chromosome exhibits a long-range (24−277 kbp) colinearity relative to other *Rickettsia* genomes, although it is more frequently interrupted by inversions/translocations than is observed between other *Rickettsia* genomes (Figure 2A). This colinearity allowed the precise assessment of orthologous relationships between ORFs of five *Rickettsia* species *(R. felis*, *R. conorii*, *R. sibirica*, *R. prowazekii*, and *R. typhi)*. On this basis, we identified 530 *R. felis*–specific ORFs, that were either absent or degraded (split or fragmented) in the other four *Rickettsia* genomes (Tables 2 and 3). Consistently, the *R. felis* genome exhibited a much higher number of gene families than other *Rickettsia* species (see Table 1). The *R. felis–*specific ORFs included a remarkably high number of paralogs for transposases, surface cell antigens *(sca)*, global metabolism regulators *(spoT)*, and proteins containing protein–protein interaction motifs such as ankyrin repeats and tetratricopeptide repeats (TPRs). Furthermore, we identified many other ORFs putatively associated with the adaptations of *R. felis* to its host environment or with its pathogenesis.

**Table 2:**
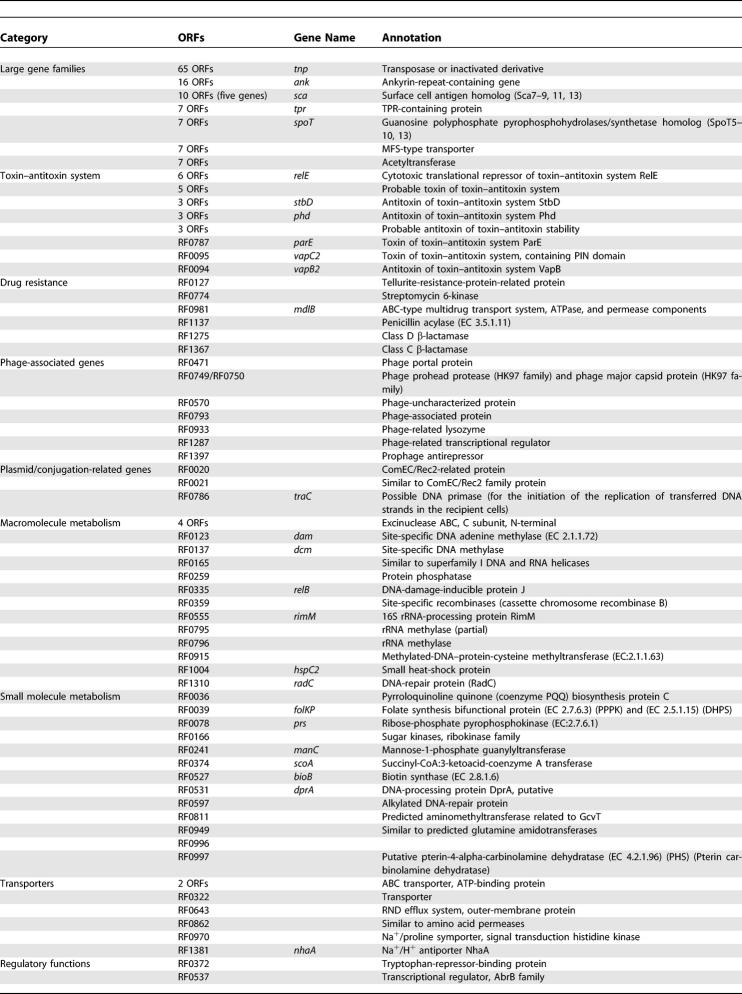
*R. felis*–Specific Genes Encoded in the Chromosome

**Table 2:**
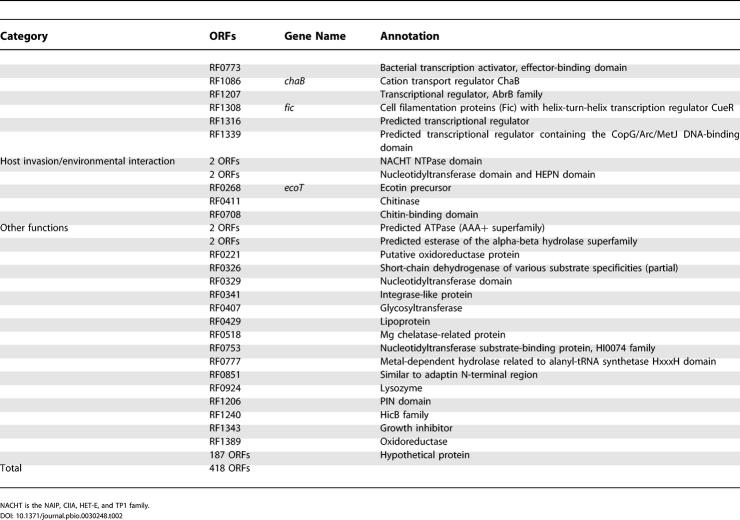
Continued

**Table 3:**
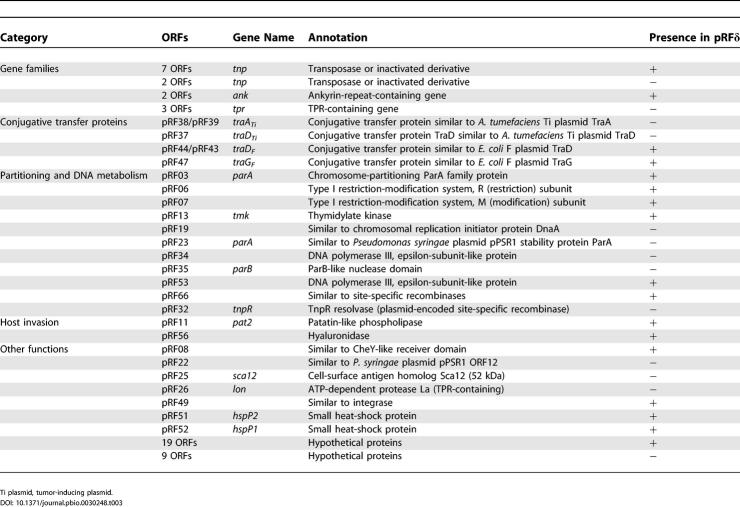
*R. felis* ORFs Encoded in pRF Plasmid

### Plasmids

The two *R. felis* plasmids, named pRF and pRFδ, are 62,829 bp and 39,263 bp long, respectively. Their topologies and sizes were confirmed experimentally (Figures S1 and S2). The pRF plasmid contains 68 ORFs, of which 53 (77.9%) exhibited homologs in public databases and 44 (64.7%) were associated with functional attributes. The nucleotide sequences of pRFδ and pRF are identical, except for an additional 23,566-bp segment that contains 24 ORFs (pRF15–pRF38) in pRF (see Table 3). These plasmids are likely to be *R. felis* specific since all attempts to detect specific plasmid sequences by polymerase chain reaction (PCR) from DNA of available reference rickettsial species were unsuccessful. In contrast, the same assays against 30 fleas naturally infected by *R. felis* resulted in amplification of the plasmid sequences in all cases.

Plasmids are referred to as conjugative or nonconjugative. The former are disseminated by conjugation from cell to cell, while the latter are only vertically transmitted. The pRF plasmid encodes several homologs of proteins involved in the different conjugative steps (see Table 3; Figure S3). First, it exhibits a split gene (pRF38/pRF39) homologous to the *traA*_Ti_ of the *Agrobacterium tumefaciens* tumor-inducing plasmid [10]. TraA_Ti_ is thought to be a DNA-processing machinery with nickase and helicase activities to generate the transfer strand from the origin of transfer *(oriT)* [10]. Second, the pRF encodes another split gene (pRF43/pRF44) homologous to the *traD*_F_ in the *Escherichia coli* F plasmid. TraD_F_ is a “coupling protein” that connects the DNA-processing machinery (and transfer strand) to the mating pair formation (Mpf) apparatus, a type IV secretion system (T4SS) [11]. Finally, pRF exhibits an ORF (pRF47) similar to TraG_F_, a protein involved in the F-pilus assembly and aggregate stabilization [12].

Despite the presence of these ORFs linked to the initiation of plasmid transfer, the pRF sequence lacks clear homologs for the proteins involved in the Mpf apparatus found in other bacteria. Nevertheless, the *R. felis* chromosome (as well as other *Rickettsia* genomes) encodes most of the components of T4SS, which are highly similar to the *vir* genes of *A. tumefaciens*. Since the *R. felis* T4SS components (*virB2* [RF1075], *virB3* [RF0087], *virB4* [RF0088], *virB6* [RF0089, RF0090, RF0091, RF0092, and RF0093], *virB8* [RF0463 and RF0465], *virB9* [RF0462 and RF0466], *virB10* [RF0467], *virB11* [RF0468], and *virD4* [RF0469]) are conserved in all sequenced *Rickettsia* genomes that lack plasmids, the primary suspected role of the *R. felis* T4SS is to translocate virulence factors to hosts. However, the T4SS of *A. tumefaciens* (*vir*) and *Legionella pneumophila* (*dot/icm*) have been shown to function both as DNA-transfer machineries and as effector translocators [13]. Thus, the *R. felis* T4SS may also promote the transfer of DNA as in *A. tumefaciens*. We also noticed that the *R. felis* chromosome exhibits a DNA primase gene (RF0786) similar to TraC found in the *E. coli* IncP plasmid. TraC initiates the replication of transferred DNA strands in the recipient cells. Finally, the *R. felis* chromosome encodes a protein (RF0020) similar to competence protein ComE3, a protein (RF0964) similar to the F-pilin acetylation protein TraX, and a split gene (RF0705/RF0706) homologous to the P-pilus assembly protein FimD. In conclusion, the presence of those putative conjugative transfer genes suggests that the *R. felis* plasmids have been acquired by conjugation and that *R. felis* may still retain the capacity of transferring plasmids.

### Genome Plasticity

We identified 333 repeated DNA sequences (50 to 2,645 bp long) in the *R. felis* genome, accounting for 4.3% of the sequence, a proportion markedly higher than in other sequenced *Rickettsia* genomes (see Table 1; Figure 2B). The major source of those repeats is the proliferation of transposase genes, for which we identified 82 copies (or inactivated derivatives). Among other obligate intracellular bacteria, only *W. pipientis* *w*Mel [14] and *Parachlamydia* sp. UWE25 [15] exhibit such a high number of large mobile genetic elements. The occurrence of highly similar transposase sequences appears to play a major role in the plasticity of the *R. felis* genome (see Figure 2A). Transposase ORFs were identified at most extremities of the *R. felis* genomic segments colinear with the *R. conorii* genome, suggesting that the *R. felis* chromosome has been rearranged many times through recombination mediated by these mobile sequences. With the use of the GRAPPA software inferring the most parsimonious genome-rearrangement scenario, we estimated at least 11 inversion events between *R. felis* and *R. conorii*. In contrast, only four inversions are required to associate more distantly related *R. conorii* and *R. prowazekii* genomes. In addition to transposases, we identified eight phage-related ORFs (see Table 2). The *R. felis* genome thus appears to have been invaded more frequently by such foreign DNAs than other *Rickettsia* species. Besides long repeats, *Rickettsia* genomes are known to contain a number of small palindromic repeats (*Rickettsia* palindromic elements [RPEs]) capable of invading both coding and noncoding regions [16]. We identified 728 RPEs in the *R. felis* genome. Of these RPEs, 85 were found within ORFs and three were found in RNA-coding genes.

The *R. felis* chromosome and plasmids share several homologs, suggesting gene exchanges between these replicons. Of 68 ORFs in pRF, 11 have a close homolog (>50% amino acid sequence identity) in the chromosome; these are seven transposases, patatin-like phospholipase (pRF11), thymidylate kinase (pRF13), and two small heat-shock proteins (pRF51 and pRF52). Among these, patatin-like proteins exhibit the most intriguing phylogeny (Figure S4). The genomes of five *Rickettsia* species *(R. prowazekii*, *R. typhi*, *R. conorii*, *R. sibirica*, and *R. felis)* exhibit chromosomal patatin-like phospholipase gene *(pat1)*. Gene organization around *pat1* is similar between these *Rickettsia*. Interestingly, a phylogenetic analysis for these Pat1 and the plasmid-encoded Pat2 indicates a close relationship between Pat1 (RF0360) and Pat2 of *R. felis*, together being an outgroup of Pat1 sequences of other *Rickettsia*, suggesting a gene replacement of the chromosomally encoded *pat1* by the plasmid-encoded *pat2* in the lineage leading to *R. felis*.

Most *R. felis* genes with orthologs in other *Rickettsia* have probably been inherited vertically from a common ancestor. On the other hand, genes without orthologs in other *Rickettsia* may have been acquired by lateral gene transfer. To test this hypothesis, we analyzed the taxonomic distribution of BLASTP best hits of *R. felis* ORFs against the nonredundant database (excluding rickettsial sequences) (Figure S5). *R. felis* ORFs with orthologs in other *Rickettsia* matched preferentially (64%) with sequences from the same taxonomic group as *R. felis* (i.e., α-proteobacteria). In contrast, the BLAST best hits for the chromosomal ORFs lacking orthologs in other *Rickettsia* were found preferentially in γ-proteobacteria (31%; 58 ORFs) and cyanobacteria (18%; 33 ORFs). The taxonomic distributions of the best matches for these two ORF sets were significantly different (*p* < 0.001; χ^2^ test). This result suggests that many *R. felis*-specific genes may originate from distantly related organisms by lateral transfer. However, methods based on nucleotide composition bias failed to identify unambiguous candidates for lateral gene acquisition in *R. felis*.

### Surface Antigens

The *sca* family is one of the largest paralogous gene families in *Rickettsia* [8]. Five *sca* members have been identified in the previously published *Rickettsia* genomes. Several Sca proteins are known to account for major antigenic differences between *Rickettsia* species [17] and may play important roles in adhesion to host cells [18]. Sca proteins are characterized by highly variable N-terminal sequences and a conserved C-terminal autotransporter β-domain, which translocates the N-terminal part outside the outer membrane. The *R. felis* genome exhibits the highest number of *sca* genes among currently available *Rickettsia* genomes. We identified nine intact *sca* paralogs *(sca1, sca2, sca3, sca4, sca5/ompB, sca8, sca9, sca12*, and *sca13)* as well as four fragmented or split paralogs *(sca0/ompA, sca7, sca10*, and *sca11)*. Reverse transcriptase–polymerase chain reaction (RT-PCR) experiments demonstrated that, under mild log growth phase, all *R. felis* *sca* paralogs were transcribed, including split ones. Phylogenetic analyses suggest that ancient duplication events gave rise to these paralogs before the divergence of *Rickettsia* species. We noticed that *sca* genes exhibit highly different patterns of presence/absence across different *Rickettsia* species (Table S1). Only *ompB* and *sca4* are conserved in all available *Rickettsia* genomes [19], remaining members being degraded or absent in one or more species. Together with the accelerated amino acid changes, differential gene degradation of *sca* paralogs probably contributes to the intra-species variation of those cell-surface proteins and might be linked with their adaptation to different host environments.

*R. felis* is genetically and serologically classified into the SFG of *Rickettsia* [20]. However, cross-reactivities caused by both proteins and lipopolysaccharides have been found with *R. typhi* using mouse sera [2] and human sera (Figure S6). *R. conorii* rarely cross-reacts with *R. typhi*. We therefore suspected that genes found in both *R. felis* and *R. typhi*, but missing in *R. conorii*, might be responsible for the cross-reactivities of *R. felis* and *R. typhi*. A list of such genes includes a *sca* family gene *(sca3)*, encoding a protein with a predicted molecular weight of 319 kDa, and *rfaJ* for the lipopolysaccharide 1,2-glucosyltransferase (Table 4).

**Table 4:**
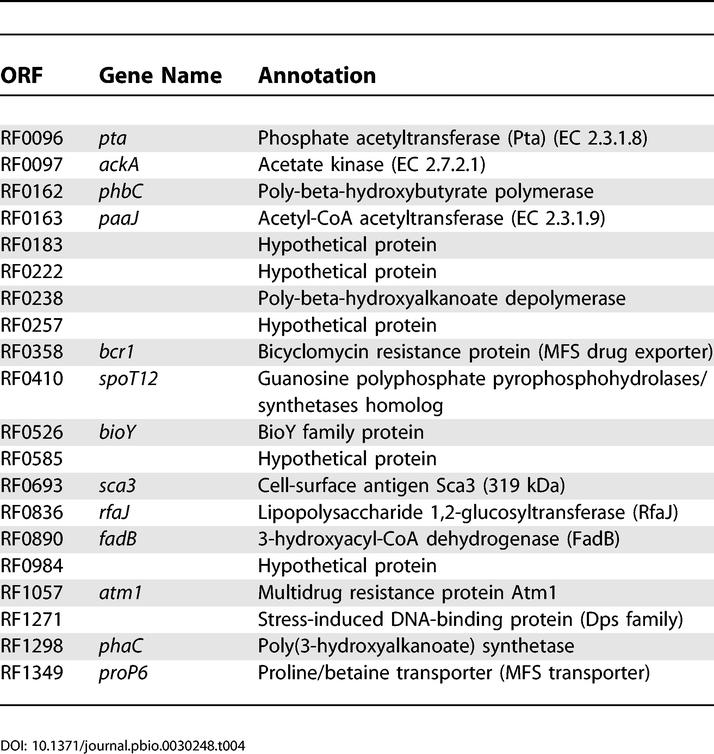
*R. felis* ORFs Present in *R. typhi* but Absent or Degraded in *R. conorii* and *R. sibirica*

### Adaptation to Environment

Transcriptional regulation may be of critical importance in *R. felis*, as the numbers of *spoT*, the gene regulating “alarmone,” and chromosomal toxin–antitoxin modules are higher in the *R. felis* genome than in any other sequenced bacterial genome.

SpoT and RelA are two hallmark enzymes regulating global cellular metabolism of *E. coli* in response to starvation [21]. These enzymes control the concentration of alarmone, (p)ppGpp (guanosine tetra- and pentaphosphates), which in turn acts as an effector of transcription. Remarkably, *R. felis* exhibits 14 *spoT*, *(spoT1–13* and *15)* paralogs (Figure S7). Using RT-PCR, we examined the transcription status of 14 *R. felis* *spoT* genes. All the *spoT* ORFs were transcribed. We classified these ORFs into two groups, based on their alignment against the sequence of the *Streptococcus dysgalactiae* Rel*_seq_* that possesses both (p)ppGpp hydrolase and synthetase activities [22]. The first group (SpoT1–10, 14, and 15) was aligned with the sequence of the hydrolase domain, and the second group (SpoT11, 12, and 13) with the sequence of the synthetase domain. Being consistent with the previous observation [23], our phylogenetic analyses suggest that each paralogous gene group originated in early duplication events before the divergence of *Rickettsia* species. Notably, every sequenced *Rickettsia* genome encodes at least one ORF exhibiting hydrolase catalytic residues and one ORF exhibiting synthetase catalytic residues, suggesting that both hydrolase and synthetase functions are required for *Rickettsia*. We also found that seven *spoT* *(spoT1–4* and *7–9)* genes were located in the *R. felis* chromosome next to a gene encoding a transporter of the major facilitator superfamily (MFS) including proline/betaine transporters. MFS is also a large paralogous gene family composed of at least 23 ORF members in *R. felis*.

Toxin–antitoxin systems are composed of tightly linked toxin and antitoxin gene pairs and ensure stable plasmid inheritance when they are encoded in plasmids. In these systems, the toxic effect of a long-lived toxin is continuously inhibited by a short-lived antitoxin only when whole systems are maintained. The toxin–antitoxin modules have also been found on the chromosomes of many free-living prokaryotes, but have rarely been found in obligate intracellular bacteria [24,25]. In the *R. felis* chromosome, we identified 16 toxin genes (RF0016, RF0095, RF0271, RF0456, RF0490, RF0602, RF0701, RF0732, RF0787, RF0792, RF0898, RF0911, RF0956, RF1272, RF1286, and RF1368) and 14 antitoxin genes (RF0015, RF0094, RF0272, RF0457, RF0489, RF0601, RF0702, RF0731, RF0779, RF0788, RF0899, RF0910, RF0957, and RF1369), comprising at least 13 modules in operon structures. It is suggested that toxin–antitoxin systems, when encoded on the bacterial chromosome, might be involved in selective killing (a primitive form of bacterial apoptosis) or reversible stasis of bacterial subpopulations during periods of starvation or other stress [26,27]. It is also tempting to speculate that the toxin–antitoxin system could be targeted to the eukaryotic host cells. In this case, this system may help to maintain the presence of bacteria in the host. Notably, in the chromosomally encoded *mazEF* system of *E. coli*, the toxin action is regulated by (p)ppGpp. The large number of toxin–antitoxin modules in *R. felis*, as well as a number of *spoT* paralogs, might thus be linked to the synchronization of its multiplication within eukaryotic hosts.

It is probable that five *R. felis*–specific ORFs are related to its capacity of antibiotic resistance. We identified a streptomycin resistance protein homolog (RF0774), a class C β-lactamase, AmpC (RF1367), a class D β-lactamase (RF1275), a penicillin acylase homolog with conserved catalytic residues (RF1137), and an ABC-type multidrug transport-system protein, MdlB (RF0981). AmpC β-lactamase is known to be induced by AmpG of the MFS, which was also identified in the *R. felis* genome (RF0265, RF0608, RF0834, and RF1247). In vivo β-lactamase activity of *R. felis* was measured using high-performance liquid chromatography (see below).

### Adaptation to Eukaryotic Hosts

*R. felis* may have developed a specific mechanism to cross-talk with its eukaryotic hosts. It exhibits 22 ankyrin-repeat-containing proteins and 11 TPR-containing proteins. These two protein motifs are frequently found in eukaryotic proteins, but their distributions are rather limited in viruses and bacteria, in both of which they appear to be linked with pathogenicity.

The ankyrin repeat is a protein–protein interaction motif, involved in transcription initiation, cell cycle regulation, cytoskeletal integrity, and cell-to-cell signaling [28]. *Anaplasma phagocytophilum*, a closely related intracellular α-proteobacterium, exhibits a protein containing ankyrin repeats (AnkA), which was detected in the cytoplasm and the nucleus of infected eukaryotic cells (human leukemia-60) [29]. According to the Superfamily database [30], only 15 bacterial species possess more than three ankyrin-repeat-containing proteins, and two species exhibiting the highest number of ankyrin repeats are obligate intracellular bacteria, *W. pipientis* (21 proteins) and *Coxiella burnetii* (20 proteins), although Wu et al. [14] reported slightly different numbers of ankyrin-repeat-containing proteins for these species. A recent genome analysis of a facultative intracellular bacterium, *L. pneumophila*, revealed 20 proteins with ankyrin repeats [31]. Ankyrin repeats were also found in more than 30 ORFs of the giant virus *Acanthamoeba polyphaga* Mimivirus [32].

TPR, composed of a motif of 34 amino acids organized in tandem, is also recruited by different proteins and facilitates protein–protein interactions [33]. Its role in the adaptation of parasites to their hosts has been suggested. The *R. felis* genome exhibits 11 TPR-containing ORFs (seven in the chromosome and four in the pRF plasmid). Only *Leptospira interrogans* (the agent of leptospirosis), *Treponema* species (including the agent of syphilis), and *L. pneumophila* [31] exhibit a high number of both TPR and ankyrin repeats. These organisms are eukaryotic parasites. The cryptococcal crooked neck 1 gene of *Cryptococcus neoformans* (a yeast), containing 16 copies of TPR, appears associated with its virulence [34].

### Host Invasion/Pathogenesis

Plasmids often carry out functions that benefit bacteria in their survival or expression of virulence. pRF exhibits two ORFs that are possibly associated with the pathogenesis of *R. felis*: a hyaluronidase and a patatin-like protein. The hyaluronidase homolog (pRF56) exhibits a significant homology to hyaluronidase NagI (1,297 aa) of *Clostridium perfringens*. Hyaluronidases, which depolymerize hyaluronic acid—an unbranched polysaccharide ubiquitously present in the extracellular matrix of animal tissues—are known as “spreading factors” [35]. Another ORF *(pat2)* exhibits a significant homology to patatin-like phospholipases. Its paralog *(pat1)* was also identified in the chromosome, as already mentioned. Patatin is the major storage glycoprotein found in potato tubers, but also exhibits phospholipase A_2_ activity for protection from infection. Proteins containing patatin-like domains are more frequently found in pathogenic than in nonpathogenic bacteria. McLeod et al. [7] suggested that patatin-like proteins might be responsible for the phospholipase A_2_ activity identified some years ago in rickettsiae [36].

Potential host-invasion capacity is also provided by *R. felis*–specific ORFs found on the chromosome, for instance, a chitinase homolog (RF0413) and a chitin-binding protein homolog (RF0710). Chitin is a homopolymer of *N*-acetylglucosamine and a major component of the exoskeleton of arthropods and of the peritrophic envelope of insects, a lining layer of the midgut. These genes may facilitate the access of bacteria to the insects' gut epithelial cells. *R. felis* may also use chitin as a nutrient source, as does *Vibrio cholerae* [37]. We identified a homolog (RF0268) for ecotin, an *E. coli* periplasmic protein inhibiting activities of a variety of proteases. Two *R. felis*–specific ORFs (RF0449 and RF0855) exhibit the complete NACHT NTPase domain. In eukaryotes, this NTPase domain has been found in proteins implicated in apoptosis as well as in immune/inflammatory responses [38]. The presence of this domain in other bacterial ORFs is limited to several lineages, such as cyanobacteria and *Streptomyces*, and their functions are unknown.

Higher eukaryotes and prokaryotes nucleotide-binding domain (HEPN) is a recently identified domain detected in a few prokaryotes. We found four genes (two were split) exhibiting HEPN at the C-terminus, and a nucleotidyl transferase domain at the N-terminus. Among other bacteria, only *A. tumefasciens*, *Thermotoga maritima*, and *Sinorhizobium melitoti* were found to exhibit HEPN-containing genes [39]. The nucleotidyl transferase domain has been associated with several classes of bacterial enzymes responsible for resistance to aminoglycosides. HEPN was also found in the human sacsin protein, a chaperonin implicated in a neurodegenerative disease. Finally, *R. felis* exhibits an ortholog (RF0371) for *R. conorii* RickA, which induces its actin-based motility [40].

### Phenotypic Post-Genomics Analysis

**Figure 3:**
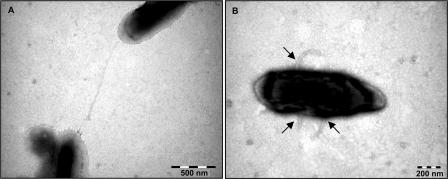
Visualization of *R. felis* Pili by Transmission Electron Microscopy Bacteria collected from the supernatant of *R. felis*–infected XTC cells were negatively stained. (A) Sexual pilus observed between two bacteria. (B) *R. felis* also possesses small appendages likely to be fimbriae pili.

The obligate intracellular nature of *R. felis* hindered progress in the detailed characterization of its phenotypic diversity. Here, we envisaged post-genomics as a way of associating in vivo phenotypes of these bacteria to genomic features. The presence of pili-associated genes prompted us to investigate, by electron microscopy, the presence of such appendages on the cell surface. This approach led to the first characterization of pili on the surface of a *Rickettsia;* we observed two forms of pili at the surfaces of *R. felis* (Figure 3). One form of pili establishes direct contact between bacteria, providing a very typical figure of Mpf apparatus; these pili are probably specialized in conjugation. The other form of pili forms small hair-like projections emerging out from the cell surface; these pili are probably involved in the attachment of the bacteria to other cells. Without pili, many disease-causing bacteria lose their invasion capability. The latter type of pili might be considered as virulence factors, as described for *Francisella tularensis* [41,42].

As previously mentioned, we also found a RickA homolog in the *R. felis* genome [40]. Based on this finding, we performed immunofluorescence assays. The orientations of actin filaments beside bacteria are distinct from the stress fibers of the host. This further suggests that *R. felis* is probably capable of using the actin cytoskeleton to disseminate through eukaryotic cells, a method exploited by other SFG rickettsiae [40] (Figure S8). Another *R. felis* phenotypic character suggested from genomic analyses (three ORFs for patatin-like proteins) was its hemolytic capacity. We confirmed experimentally that *R. felis* lyses erythrocytes, this effect being inhibited by dithiothreitol. Another genome-guided discovery was β-lactam inhibition, which reached 57% and 53% of the concentration and the minimal inhibitory concentration, respectively, following 2 h incubation of *R. felis* with amoxicillin. Despite being preliminary results, these findings illustrate the fact that whole-genome sequencing offers opportunities to rapidly gain a better understanding of the phenotypic characters of a fastidious microorganism.

## Discussion

*R. felis* is the first obligate intracellular bacterium exhibiting a possible conjugative plasmid. Of the nine previously published studies of members of the Order Rickettsiales (six in Rickettsiaceae, three in Anaplasmateceae), none exhibited a plasmid. Several other obligate intracellular bacteria, such as *Chlamydia muridarum, Chlamydophila caviae, C. burnetii, Wigglesworthia glossinidia*, and *Buchnera aphidicola*, are known to possess plasmids. Recently, the reannotation of the genome of *Parachlamydia*, an obligate intracellular bacteria living in amoeba, predicted an F-like DNA conjugative system encoded in a genomic island [43]. However, no conjugation has yet been observed for those plasmids and genomic island. Transformation of obligate intracellular bacteria remains an elusive goal, although preliminary work on several obligate intracellular bacteria has been reported with limited results [44]. The possible conjugative plasmid identified in *R. felis* may provide a molecular basis for the future development of new genetic transformation tools in rickettsiae.

*R. felis* is hosted by fleas, as are *R. typhi, B. henselae*, *W. pipientis*, and *Yersinia pestis*. There are surprisingly few common genomic features between *R. typhi* and *R. felis*. *R. typhi* genetically resembles *R. prowazekii* despite having a lifestyle similar to that of *R. felis* (Table S2). The comparison with *W.* *pipientis* is interesting. This intracellular bacterium also multiplies in arthropods (including fleas) and is transmitted transovarially. The most relevant finding in its genome was the detection of repetitive mobile DNA elements. Many ankyrin repeats and several TPRs were also found. It appears that *R. felis* and *W.* *pipientis* share common genomic features, possibly because of their similar niches (we found two *Ct. felis* fleas in France coinfected with *W. pipientis* and *R. felis*). They both differ significantly from their immediate neighbors [45,46]. Moreover, the phylogenetic relationship and hosts of *R. felis* and *R. prowazekii* (transmitted by lice) are comparable with those for *B. quintana* (transmitted by lice) and *B. henselae* (transmitted by fleas) [47]. *B. henselae* exhibits a larger genome with more repeats and integrases than *B. quintana*. *Y. pestis*, transmitted by fleas, also exhibits many more insertion sequences than its close relative, *Y. pseudotuberculosis* [48]. Altogether, flea-infecting bacteria appear to exhibit a specific evolution (i.e., more repeats, transposases, and/or integrases) compared with their non-flea-infecting neighbors.

For obligate intracellular bacteria such as rickettsiae, few phenotypic characters have been observed. To date, four intracellular bacterial genomes have been entirely sequenced, the procedure being completed in 7 y or less after their first identification or culture, including *R. felis* [14,15,49,50]. In the present study, the genome sequencing of *R. felis* provided evidence of the presence of conjugative plasmids, two types of pili, hemolytic activity, β-lactamase activity, and intracellular motility. We believe that for such recently identified/cultured fastidious organisms, complete genome sequencing is a very potent and timesaving strategy to identify unrecognized phenotypic properties.

## Materials and Methods

### Bacterial purification and DNA extraction

*R. felis* (strain California 2) was cultivated on XTC cells growing on RPMI with 5% fetal bovine serum, supplemented with 5 mM L-glutamine. The purification of the bacteria was performed by different steps. First, the bacteria were treated in the presence of 1% trypsine in K36 buffer for 1 h at 37 °C, then centrifuged and digested by DNAseI for 1 h at 37 °C to reduce the eukaryotic DNA contamination. The sample was loaded on a renograffin gradient and the bands of the purified bacteria were washed in K36, treated again by DNAseI. After inactivation with EDTA (50 mM), the bacteria were resuspended in TE, dispatched in 150-μl tubes and stored at −80 °C. Depending on this initial concentration, one or two tubes were diluted in 1 ml of TNE (10 mM Tris [pH 7.5], 150 mM NaCl, 2 mM EDTA) and incubated for 5 h at 37 °C in the presence of lysozyme (2 mg/ml). Lysis was performed for 2 h at 37 °C by adding 1% SDS and RNAseI (25 μg/ml). Overnight treatment with 1 mg/ml of proteinase K followed at 37 °C. After three phenol–chloroform extractions and alcoholic precipitation, the DNA was resuspended in 30 μl of TE and its concentration was estimated by agarose gel electrophoresis.

### Pulsed-field agarose gel electrophoresis

The concentrated bacterial suspension was included in 1% (vol/vol) Incert agarose gel blocks (BMA, Rockland, Maryland, United States). The agarose blocks were digested by Proteinase K (1 mg/ml) (Eurobio Laboratories, Paris, France) in 1% lauroylsarcosine and 0.5 M EDTA (pH 8) (Sigma-Aldrich, St. Louis, Missouri, United States) for 24 h at 50 °C. Fresh Proteinase K was then added and the incubation was continued for 24 h. The blocks were then washed twice in TE (pH 7.6) for 30 min at room temperature. Proteinase K inactivation was performed through incubation in a 4% phenylmethylsulfonyl fluoride (MBI Fermentas, Burlington, Canada) solution for 1 h at 50 °C. This inactivation step was carried out twice. The blocks were then washed two to three times in TE and stored in 0.5 M EDTA (pH 8) at 4 °C. Before restriction enzyme digestion, the agarose blocks were equilibrated twice with TE for 15 min. Digestion was carried out for 4 h, then fresh enzyme was added and the incubation was continued overnight. The digested agarose blocks and molecular-weight markers (Low Range PFG Marker, Lambda Ladder PFG Marker [New England Biolabs, Beverly, Massachusetts, United States]) were equilibrated in 0.5× TBE (50 mM Tris, 50 mM boric acid, 1 mM EDTA).

Each agarose block was laid in a 1% PFEG agarose (Sigma-Aldrich) solution in 0.5× TBE. Pulsed-field gel electrophoresis was carried out on a CHEF-DR II device (Bio-Rad, Hercules, California, United States) under different electrophoresis conditions. The 1% agarose gel was run at 200 V using ramped pulse times from 1 to 5 s for 10 h to observe the pattern of small DNA fragments (2–48 kb). The migration was taking place under the following two consecutive conditions: (i) a ramping time from 3 to 10 s at 200 V for 12 h, with the pattern representative for 48- to 242-kb fragments, then (ii) a ramping time from 20 to 40 s at 180 V for 15 h, with the pattern representative for 145- to 610-kb fragments.

### Shotgun of *R. felis* genome and sequencing strategy

Three shotgun genomic libraries were constructed by mechanical shearing of the genomic DNA using a Hydroshear device (GeneMachine, http://genome.nhgri.nih.gov/genemachine/). DNA fragments were blunt-ended using T4 DNA polymerase (New England Biolabs) and ligated to the BstXI adapter. Fragments of 3, 4.5, and 7 kb were separated on a preparative agarose gel (FMC BioProducts, Rockland, Maryland, United States), extracted with Qiaquick kit (Qiagen, Valencia, California, United States), and ligated into pCDNA2.1 (Invitrogen, Carlsbad, California, United States) for the two smaller inserts and into pCNS (a low copy number vector; C. R., unpublished data) for the largest one. DNA cloning was performed using electrocompetent *E. coli* DH10B Electromax cells (Invitrogen). Plasmid DNAs were purified and pools of 96 clones were analyzed by gel electrophoresis to validate the libraries. DNA sequencing of insert ends was carried out using Big Dye 3.1 terminator chemistry on an automated capillary ABI3700 sequencer (Applied Biosystems, Foster City, California, United States).

Sequences were analyzed and assembled into contigs using Phred, Phrap, and Consed software [51] taking all sequences into account. Sequences were considered valid when at least 75% of the nucleotides had a Phred score of more than 20. The finishing of the genome sequencing included only additional directed reactions that were performed on an ABI3100 sequencer. Two circular plasmid molecules of 63 and 38 kbp, respectively, were identified from the assembled sequences. On the chromosome, three small regions of 41, 155, and 64 bp failed by dropping of sequence. A number of parameters (DMSO, glycerol, hybridization, and elongation temperature) were tested one by one or were combined to sequence over these gaps. We finally succeeded with the association of another type of chemistry, D-rhodamine with 2 M betaine. We designed and used 420 primers (i) to close the sequencing gaps by walking either on shotgun subclones or on the chromosome and (ii) to improve sequence regions of low quality.

The integrity of the assembly was validated by comparing the restriction patterns obtained by pulsed-field gel electrophoresis with those deduced from the electronic consensus sequence. The selection of restriction enzymes was based on rare sites. We analyzed single digests of *R. felis* DNA. The main restriction enzymes used for these studies were ApaI, AfeI, FspI, and SbfI. This comparative study confirmed the predicted length of the *R. felis* DNA fragments.

The structures for pRF and pRFδ plasmids were controlled by specific primer amplifications (see Figure S1). Three PCRs were performed and the amplification results were in agreement with the expected hypothesis. These PCR results validate the two distinct plasmid forms (62.8 and 39 kbp, respectively). Meanwhile, a Southern blot was performed through a pulsed-field electrophoresis gel. Uncut genomic *R. felis* DNA and *R. felis* DNA digested by the restriction enzyme PvuI (corresponding to a unique site in the pRF-specific region) were analyzed. These blocks of DNA were loaded twice onto the gel with the molecular-weight markers: Lambda Marker (Bio-Rad) and Low Range PFG Marker (New England Biolabs) as described above, with a pulse time from 1 to 5 s for 12 h at 180 V. The gel was treated and transferred onto Hybond N+ (Amersham Biosciences, Little Chalfont, United Kingdom) with a vacuum blot. The DNA was fixed by heating for 2 h at 80 °C, and the membrane was cut into two pieces. Two probes were derived from two PCR products. The first, pRFh–pRFi (726 bp), was designed within the pRF-specific insert, and the second, pRFa–pRFg (251 bp), was designed to encompass the deletion site of the pRFδ. These two probes were labeled with dCTP^32^ and hybridized at 65 °C for 17 h on each membrane. Membranes were washed three times in 1× SSC and 0.1% SDS at 65 °C. The exposure time ranged from 6 h to overnight at −80 °C on ECL film. The hybridizations were clearly established on *R. felis* digested by PvuI and led to one signal with the pRFh–pRFi probe and two signals for the two plasmid structures with the pRFa–pRFg probe at a predicted molecular weight compatible with our prediction (see Figure S2).

We tested 30 samples of fleas naturally infected by *R. felis* obtained from different geographic areas (Algeria [11 fleas], France [15 fleas], and New Zealand [four fleas]) with three pairs of primers: (i) primers designed in the *traD* gene (pRF37F1/R1), (ii) primers in the pRF plasmid (pRFe–pRFf), and (iii) primers in the pRFδ plasmid (pRFa–pRFg). We confirmed positive PCR products of (i) 196 bp, (ii) 208 bp, and (iii) 251 bp for all the 30 cases.

### Annotation

We predicted protein-coding genes (ORFs) using SelfID [52] as previously described [8]. tRNA genes were identified using tRNAscan-SE [53]. Database searches were performed using BLAST programs [54] against Swiss-Prot/TrEMBL [55], the NCBI CDD database [56], and SMART [57]. The number of transposases, ankyrin/TPR-containing genes, autotransporter domains, and integrases were computed using PSI-BLAST with NCBI/CDD entries related to those domains with an *E*-value threshold of 10^−5^. Repeated DNA sequences were identified with the use of RepeatFinder [58], by ignoring the sequence similarity between pRF and pRFδ. To identify *Rickettsia* palindromic elements, we used hidden Markov models [59] based on the previously identified RPE sequences [60].

By taking advantage of genome colinearity, we identified orthologous relationships of genes in *R. felis, R. conorii, R. sibirica, R. prowazekii*, and *R. typhi* with the use of Genomeview (S. Audic, unpublished software). Based on the gene orthology, we defined *R. felis*–specific ORFs, which were of one of the following three classes: Class I ORFs exhibiting no homologous ORFs in the other four *Rickettsia* genomes; Class II ORFs exhibiting homologous ORFs but no orthologous ORFs in the other four *Rickettsia* genomes; and Class III ORFs exhibiting orthologous ORFs in some or all of the other four *Rickettsia*, all of which exhibit degraded (split or fragmented) genes relative to the *R. felis* ORF. Plasmid-encoded ORFs were by definition classified into Class I or II. A gene composed of more than one ORF was defined as “split gene.” A gene composed of a single ORF whose length is shorter than 50% of the longest ortholog was defined as a “fragmented” ORF. We used T-Coffee [61] and MEGA [62] for multiple sequence alignment and phylogenetic tree analyses, respectively. The analyses of horizontal gene transfer were performed by BLAST search against the Swiss-Prot/TrEMBL nonredundant database, excluding rickettsial sequences, as well as by methods based on nucleotide composition bias [63,64]. We obtained the minimum number of inversions to associate a pair of *Rickettsia* genomes using GRAPPA release 2.0 [65].

### Ultrastructural characterization of pili by electronic microscopy

*R. felis* cells were carefully collected from the supernatant of XTC cells infected for 5 d and grown at 28 °C. Following centrifugation (400 *g*, 10 min), bacteria were fixed for 1 h at 4 °C in glutaraldehyde (2.5% in phosphate-buffered saline [PBS]). Cells were then washed in PBS and placed on a carbon–formvar-coated 400-mesh copper grid (Electron Microscopy Sciences, Hatfield, Pennsylvania, United States) for 15 min then negatively stained with 2% phosphotungstic acid for 10 s, before analysis by electron microscopy (Philips Morgagni 268D, Philips Electronics, Eindhoven, the Netherlands).

### Estimation of β-lactamase activity

To evaluate the level of β-lactamase activity, 10^4^ *R. felis* cells grown on XTC cells and then sonicated were mixed with amoxicillin to a final concentration of 20 μg/ml, and incubated for 2 h at 28 °C. The concentration of amoxicillin was measured in the *R. felis* + amoxicillin suspension as well as in a suspension of XTC cells without bacteria + amoxicillin, before and after incubation, using high-performance liquid chromatography. In addition, the minimum inhibitory concentrations of these four suspensions were estimated by growth inhibition of a *Micrococcus luteus* strain.

### RNA extraction and RT-PCR

Approximately 6.5 × 10^5^ bacteria were used to infect one 25-cm^3^ flask of confluent XTC cells maintained at 28 °C. Infected cells were harvested 48 h later, centrifuged (12,000 *g*, 10 min), and pellets were immediately frozen in liquid nitrogen before being stored at −80 °C. Total RNA was isolated by using the RNeasy Mini Kit (Qiagen) according to the manufacturer's instructions. At the end of the extraction procedure, all samples were treated with RNase-Free DNase Set (Qiagen) for 30 min. The concentration and quality of isolated RNA were determined with the Agilent 2100 bioanalyzer (Agilent Technologies, Englewood, New Jersey, United States). Aliquots of the DNase-treated total RNA samples were stored at −80 °C until use. RT-PCR was performed from 2 μl of RNA (25 μl final reaction volume) with the Superscript One-Step RT-PCR with Platinum *Taq* (Invitrogen). Possible DNA contamination was assessed with the Expand high-fidelity polymerase (Roche, Basel, Switzerland). Cycling conditions were 30 min at 50 °C, 5 min at 95 °C, and 40 cycles at 30 s at 95 °C, 30s at 50 °C, and 1 min at 72 °C, followed by a final extension cycle of 7 min at 72 °C. The RT-PCRs were conducted on the PTC-100 thermocycler (Bio-Rad). Amplification products were run on 2% (wt/vol) agarose gels, and the DNA was stained with ethidium bromide. The size of the PCR product was determined by comparison with DNA molecular-weight marker VI (Boehringer Ingelheim, Ingelheim, Germany).

### Detection of F-actin and immunofluorescence staining

Vero cells grown to semiconfluence on glass coverslips were infected with *R. felis* for 24–48 h at 28 °C in a humidified CO_2_ incubator (5% CO_2_). Infected cells were then fixed for 1 h at 4 °C with formaldehyde (3% wt/vol in PBS supplemented with 1 mM MgCl_2_ and 1 mM CaCl_2_), washed three times in PBS, and then made permeable with 0.2% Triton X-100 in PBS for 1 min. After three washings in PBS, the coverslips were incubated for 1 h with a monoclonal anti–*R. felis* antibody. Bacteria were visualized by staining with anti-mouse-Alexa 594 antibody (1:300) and F-actin with FITC-phalloidin (1:250). The coverslips were mounted using Fluoprep (BioMérieux, Marcy-l'Etoile, France) and were examined with a confocal laser scanning microscope using a 100× oil immersion objective lens.

### Hemolysis experiments

Human blood (10 ml) was centrifuged (1,500 *g*, 10 min), and after three PBS washings, erythrocytes were resuspended in 20 ml of PBS. This suspension (100 μl) was mixed with 800 μl of PBS and 100 μl of rickettsial suspension (10^6^, 10^5^, and 10^4^ bacteria, respectively). In some experiments, rickettsiae were incubated for 1 h at 35 °C in the presence of 2 mM DTT. Complete hemolysis was determined by adding 900 μl of H_2_O to erythrocytes, and spontaneous hemolysis corresponded to control without bacteria. Following 3 h of incubation at 35 °C, the samples were fixed using paraformaldehyde (0.3% final concentration) and centrifuged. Hemoglobin release was estimated by measurement of the optical density of the supernatant at 545 nm. This experiment was performed in duplicate.

### Primers

The sequences of the primers for PCR and RT-PCR are provided in Table S3.

## Supporting Information

Figure S1 Confirmation of Plasmid Topologies for pRF and pRFδ by PCR (A) The locations of the three primer sets (pRFa–pRFb, pRFc–pRFd, and pRFa–pRFd) used to validate the presence of the two distinct plasmid forms are indicated.
(B) The result of the PCR assay with these primers. Two pairs of primers (pRFa–pRFg and pRfh–pRFi) used to obtain the probes for the Southern blot (see Figure S2), as well as another pair of primers (pRF37F1/R1) used in plasmid detection in fleas infected by *R. felis*, are also indicated in (A).
(1.5 MB TIF).

Figure S2 Characterization of *R. felis* Plasmids by Southern Blot The two membranes (A and B) loaded with *R. felis* genomic DNA (#1/4) and *R. felis* DNA digested by PvuI (#2/3) were hybridized either by the probe pRFa–pRFg or by pRFh–pRFi.
(39 KB PDF).

Figure S3 A Model for the Conjugative Plasmid Transfer of *R. felis*
This model is based on gram-negative bacterial conjugation systems involving T4SS. Homologs responsible for different steps of conjugation were identified in the *R. felis* genome. DNA-processing machinery, plasmid-encoded pRF38/39 (TraA_Ti_); the coupling protein, plasmid-encoded pRF43/44 (TraD_F_) or chromosomally encoded *virD4* (RF0469); Mpf apparatus, chromosomal genes for *virB2* (RF1075), *virB3* (RF0087), *virB4* (RF0088), *virB6* (RF0089, RF0090, RF0091, RF0092, and RF0093), *virB8* (RF0463 and RF0465), *virB9* (RF0462 and RF0466), *virB10* (RF0467), and *virB11* (RF0468); priming for DNA replication in the recipient cell, chromosomally encoded RF0786 (TraC).
(57 KB PDF).

Figure S4 Phylogenetic Trees for the Patatin-Like Proteins, Thymidylate Kinases, and Small Heat-Shock Proteins Phylogenetic trees were constructed using the neighbor-joining method with Jones-Taylor-Thornton model.
(16 KB PDF).

Figure S5 Taxonomic Distribution of BLAST Best Hits of *R. felis* ORFs *R. felis* ORFs were searched against the nonredundant database (excluding rickettsial sequences). The distribution difference between ORFs with rickettsial orthologs and ORFs lacking rickettsial orthologs remained significant even after the removal of transposase ORFs.
(16 KB PDF).

Figure S6 Cross-Reactivity of *R. felis*
Western immunoblot showing the preferential cross-reactivity of antibodies with *R. felis* and *R. typhi* in a patient with murine typhus (lanes a–c), and with *R. felis* and *R. conorii* in a patient with Mediterranean spotted fever (lanes d–f). Lanes a and d, *R. conorii* antigen; lanes b and e, *R. typhi* antigen; lanes c and f, *R. felis* antigen; MM, molecular mass.
(30 KB PDF).

Figure S7 Domain Structures and the Presence/Absence Patterns of *spoT* Genes in Different *Rickettsia*
With reference to the *S. dysgalactiae* Rel_seq_, four (H: 53H, 77H, 78D, and 144D) and five (241R, 243K, 251K, 264D, and 323E) catalytic residues were examined for the (p)ppGpp hydrolase and synthetase domains, respectively. ORF sizes were those for *R. felis* genes, except SpoT14, for which the *R. prowazekii* ORF size is indicated. a, absent; Ch, conserved hydrolase catalytic residues; Cs, conserved synthetase catalytic residues; s, split or fragmented genes.
(17 KB PDF).

Figure S8 Confocal Laser Analysis of *R. felis*–Infected Vero Cells Bacteria were stained by indirect immunofluorescence using a monoclonal anti–*R. felis* antibody followed by an anti-mouse-Alexa 594 antibody (red). F-actin was stained with FITC-phalloidin (green). Arrows indicate *R. felis* with actin tail.
(29 KB PDF).

Table S1 Distribution of *sca* Genes among *Rickettsia* Genomes (30 KB DOC).

Table S2 Comparison of Different Features of Bacteria Infecting Fleas with Their Close Relatives (32 KB DOC).

Table S3 Nucleotide Sequences of the Primers Used in the Present Study (28 KB DOC).

### Accession Numbers

The genome sequence of *R. felis* is accessible via GenBank (http://www.ncbi.nlm.nih.gov/Genbank) under the accession numbers: CP000053, CP000054, and CP000055. The EMBL Nucleotide Sequence Database (http://www.ebi.ac.uk/embl) accession number for *Cl. perfringens* NagI is Q8XM09. The Pfam (http://www.sanger.ac.uk/Software/Pfam/) accession number for ComE3 is PF03772. The Protein Data Bank (http://www.rcsb.org/pdb/) accession number for *S. dysgalactiae* Rel_seq_ is 1VJ7.

## Abbreviations

HEPN: higher eukaryotes and prokaryotes nucleotide-binding domain
MFS: major facilitator superfamily
Mpf: mating pair formation
ORF: open reading frame
PBS: phosphate-buffered saline
PCR: polymerase chain reaction
(p)ppGpp: guanosine tetra- and pentaphosphates
RPE: *Rickettsia* palindromic element
RT-PCR: reverse transcriptase–polymerase chain reaction
SFG: spotted-fever group
TPR: tetratricopeptide repeat
T4SS: type IV secretion system
XTC cell: *Xenopus laevis* tissue culture cell
