# Supplementary material for: The Genome Sequence of Rickettsia felis Identifies the First Putative Conjugative Plasmid in an Obligate Intracellular Parasite
Source: PLoS Biol. 2005 Jul 5;3(8):e248. doi: 10.1371/journal.pbio.0030248 (PMC1166351; doi:10.1371/journal.pbio.0030248)
Supplement: Supplementary file 1 [file pbio.0030248.sg002.pdf]

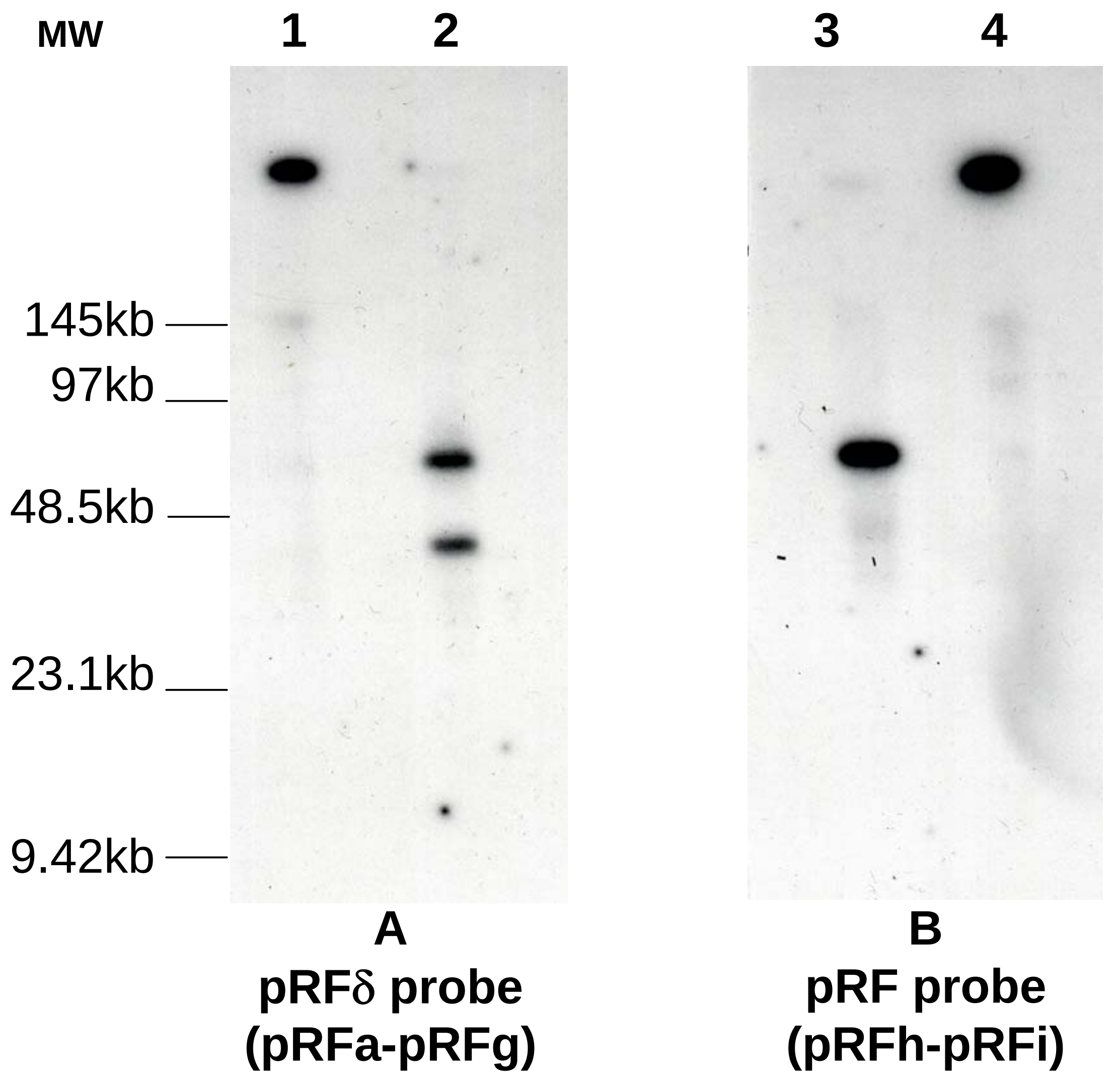
MW
1
2
3
4
145kb
97kb
48.5kb
23.1kb
9.42kb
A
pRFδ probe
(pRFa-pRFg)
B
pRF probe
(pRFh-pRFi)
