# Supplementary material for: The Genome Sequence of Rickettsia felis Identifies the First Putative Conjugative Plasmid in an Obligate Intracellular Parasite
Source: PLoS Biol. 2005 Jul 5;3(8):e248. doi: 10.1371/journal.pbio.0030248 (PMC1166351; doi:10.1371/journal.pbio.0030248)
Supplement: Supplementary file 2 [file pbio.0030248.sg003.pdf]

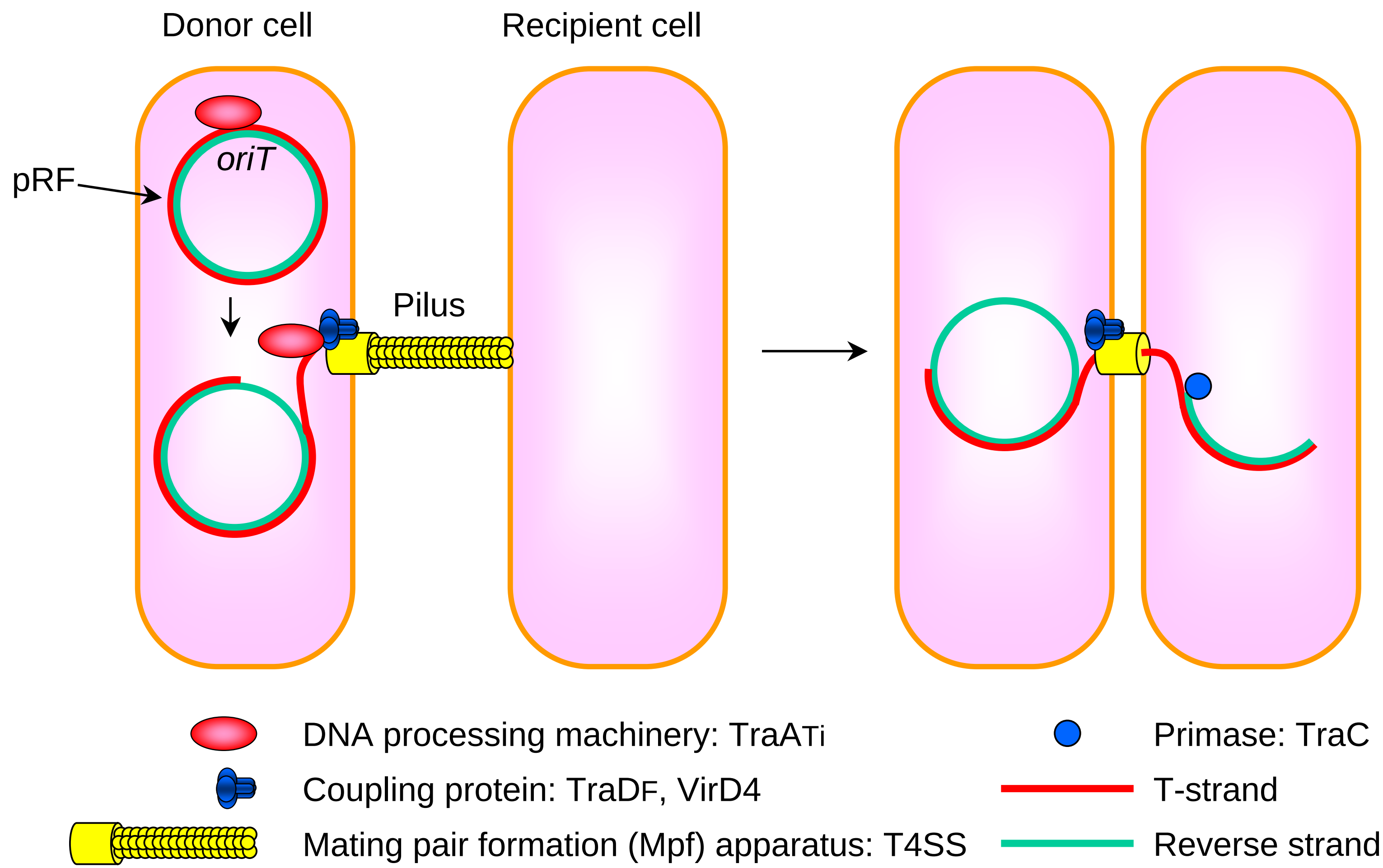

Donor cell
Recipient cell
pRF
oriT
Pilus
DNA processing machinery: TraATi
Coupling protein: TraDF, VirD4
Mating pair formation (Mpf) apparatus: T4SS
Primase: TraC
T-strand
Reverse strand
