# Supplementary material for: The Genome Sequence of Rickettsia felis Identifies the First Putative Conjugative Plasmid in an Obligate Intracellular Parasite
Source: PLoS Biol. 2005 Jul 5;3(8):e248. doi: 10.1371/journal.pbio.0030248 (PMC1166351; doi:10.1371/journal.pbio.0030248)

Patatin-like protein

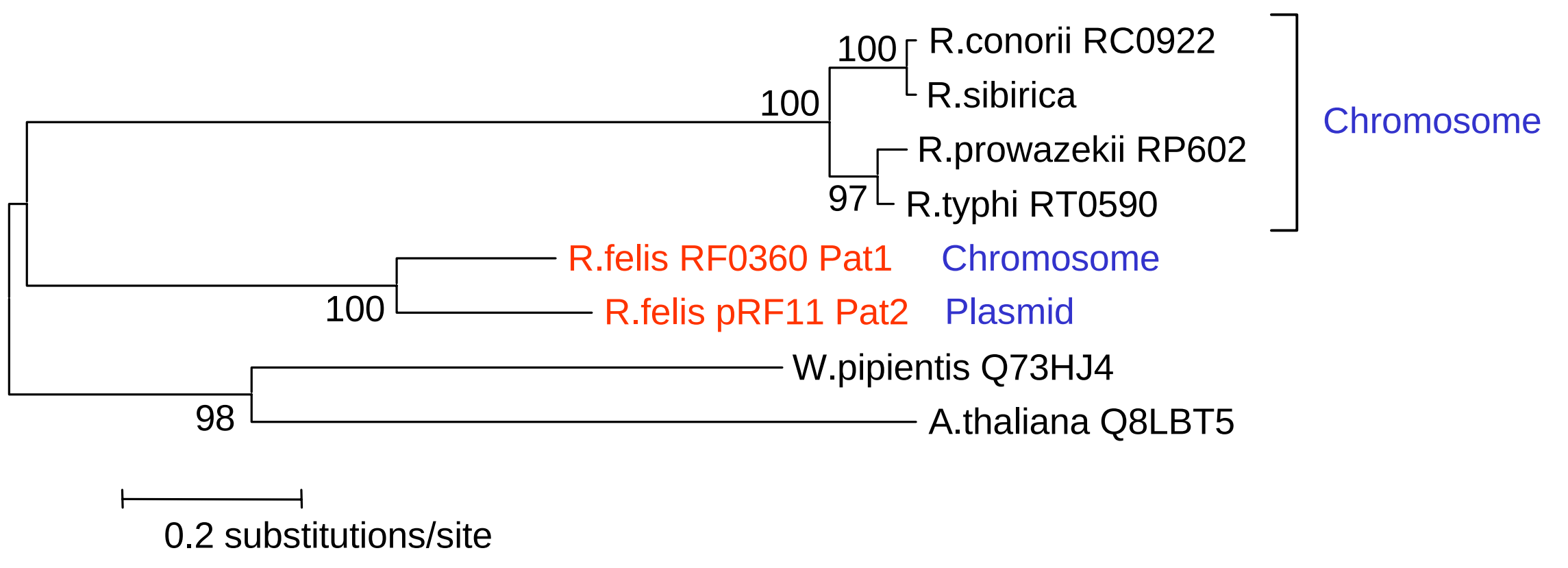

TMK

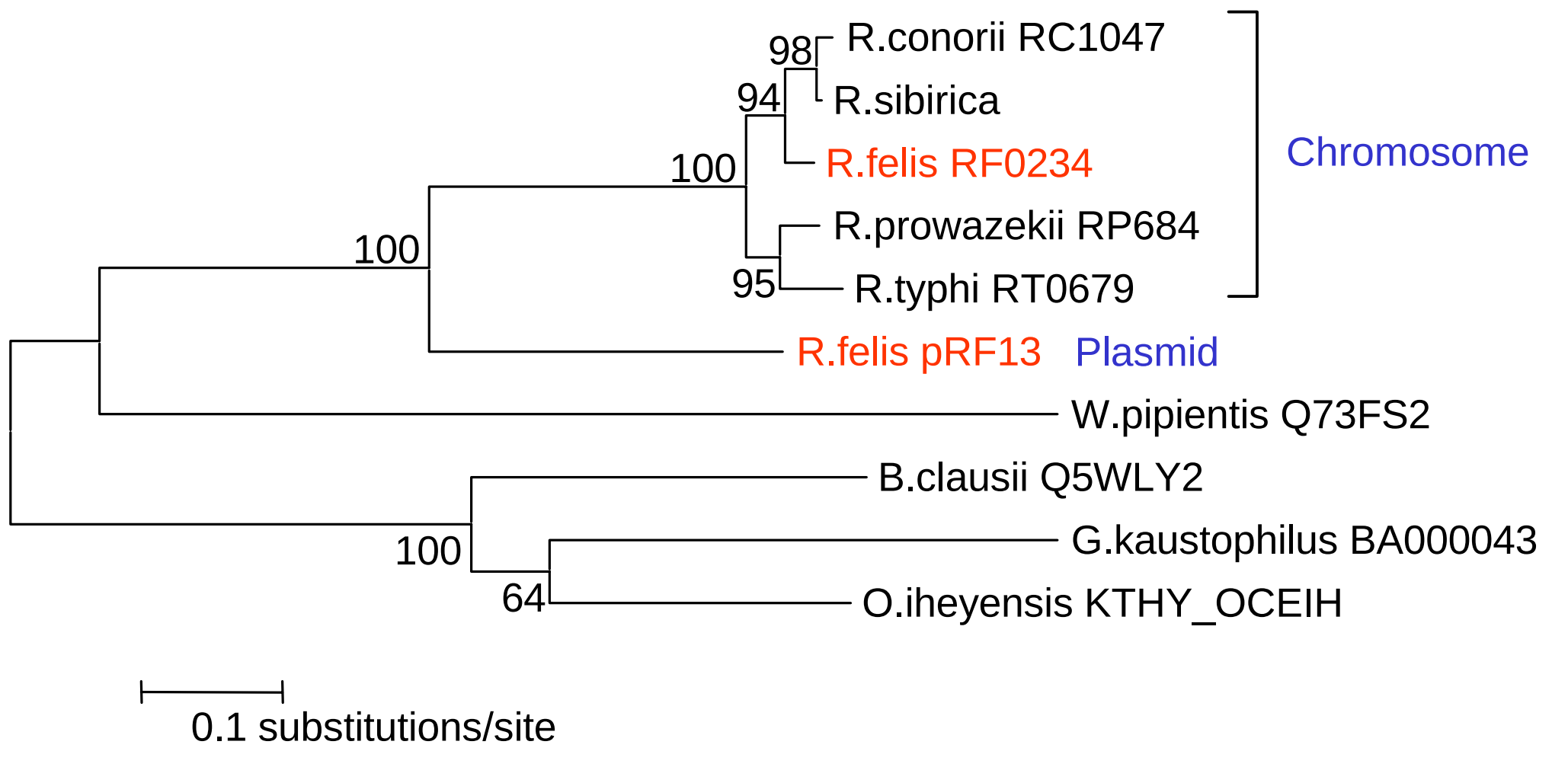

Small heat shock protein

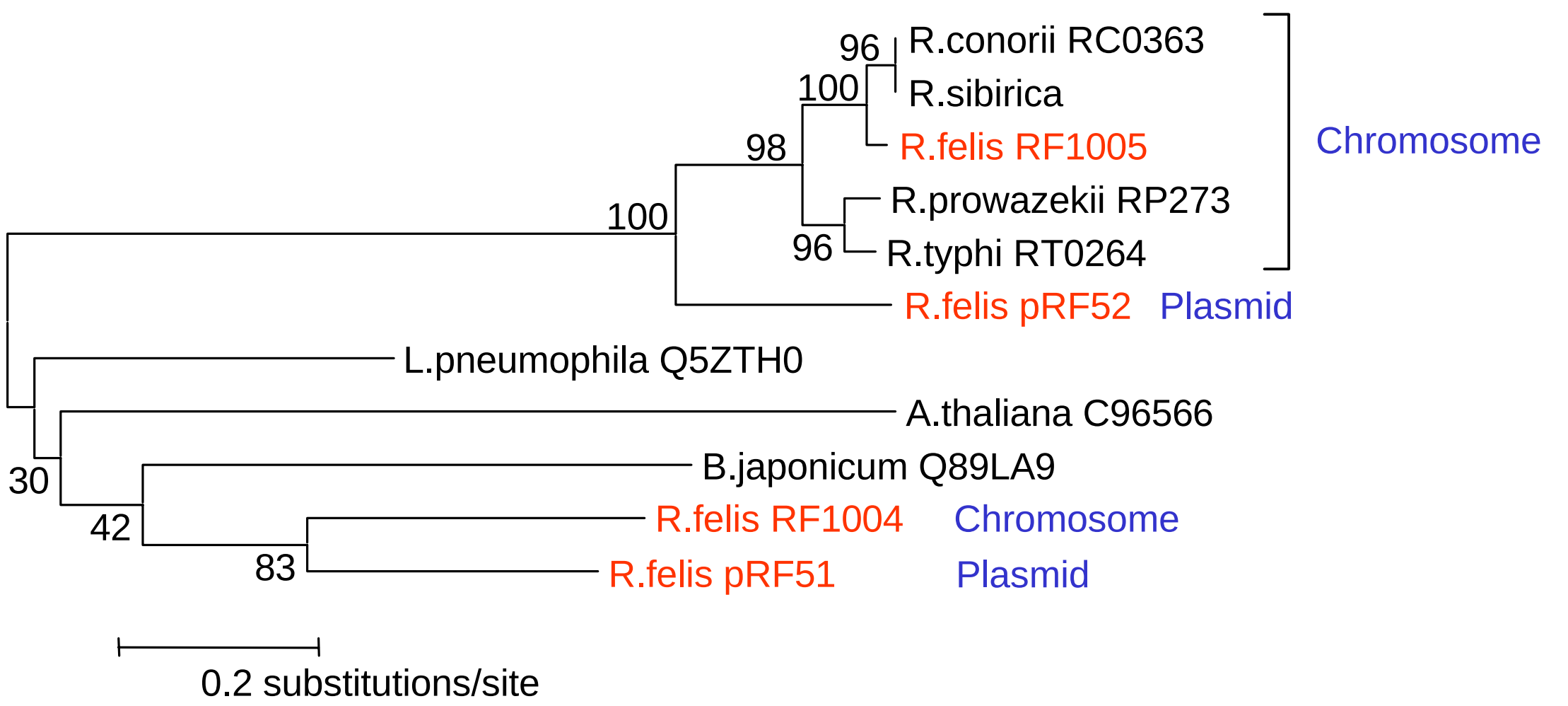

Supplement: Supplementary file 3 [file pbio.0030248.sg004.pdf]
