# Supplementary material for: The Genome Sequence of Rickettsia felis Identifies the First Putative Conjugative Plasmid in an Obligate Intracellular Parasite
Source: PLoS Biol. 2005 Jul 5;3(8):e248. doi: 10.1371/journal.pbio.0030248 (PMC1166351; doi:10.1371/journal.pbio.0030248)
Supplement: Supplementary file 4 [file pbio.0030248.sg005.pdf]

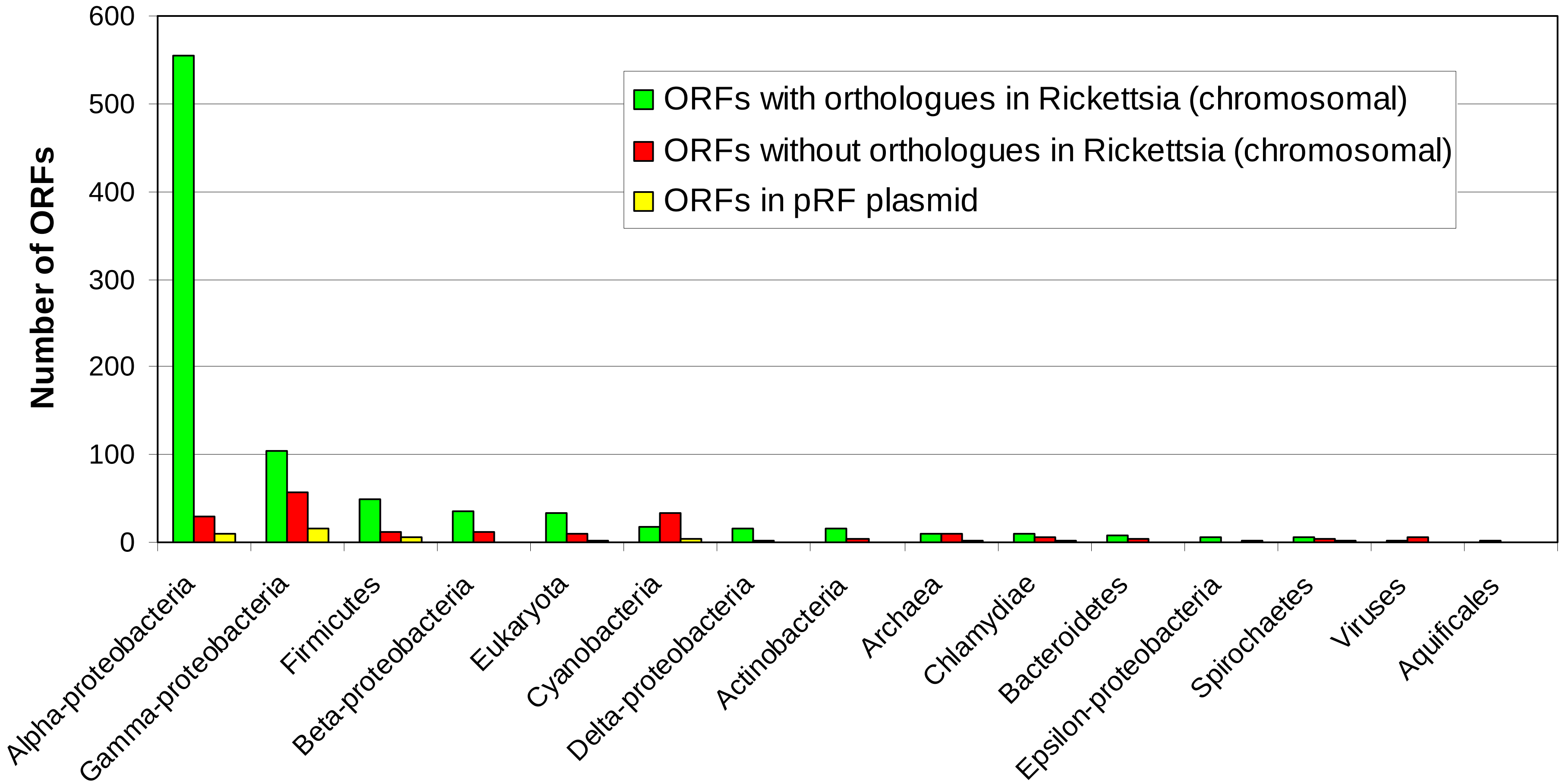
Number of ORFs
600
500
400
300
200
100
0
ORFs with orthologues in Rickettsia (chromosomal)
ORFs without orthologues in Rickettsia (chromosomal)
ORFs in pRF plasmid
Alpha-proteobacteria
Gamma-proteobacteria
Firmicutes
Beta-proteobacteria
Eukaryota
Cyanobacteria
Delta-proteobacteria
Actinobacteria
Archaea
Chlamydiae
Bacteroidetes
Epsilon-proteobacteria
Spirochaetes
Viruses
Aquificales
