# Supplementary material for: The Genome Sequence of Rickettsia felis Identifies the First Putative Conjugative Plasmid in an Obligate Intracellular Parasite
Source: PLoS Biol. 2005 Jul 5;3(8):e248. doi: 10.1371/journal.pbio.0030248 (PMC1166351; doi:10.1371/journal.pbio.0030248)
Supplement: Supplementary file 5 [file pbio.0030248.sg006.pdf]

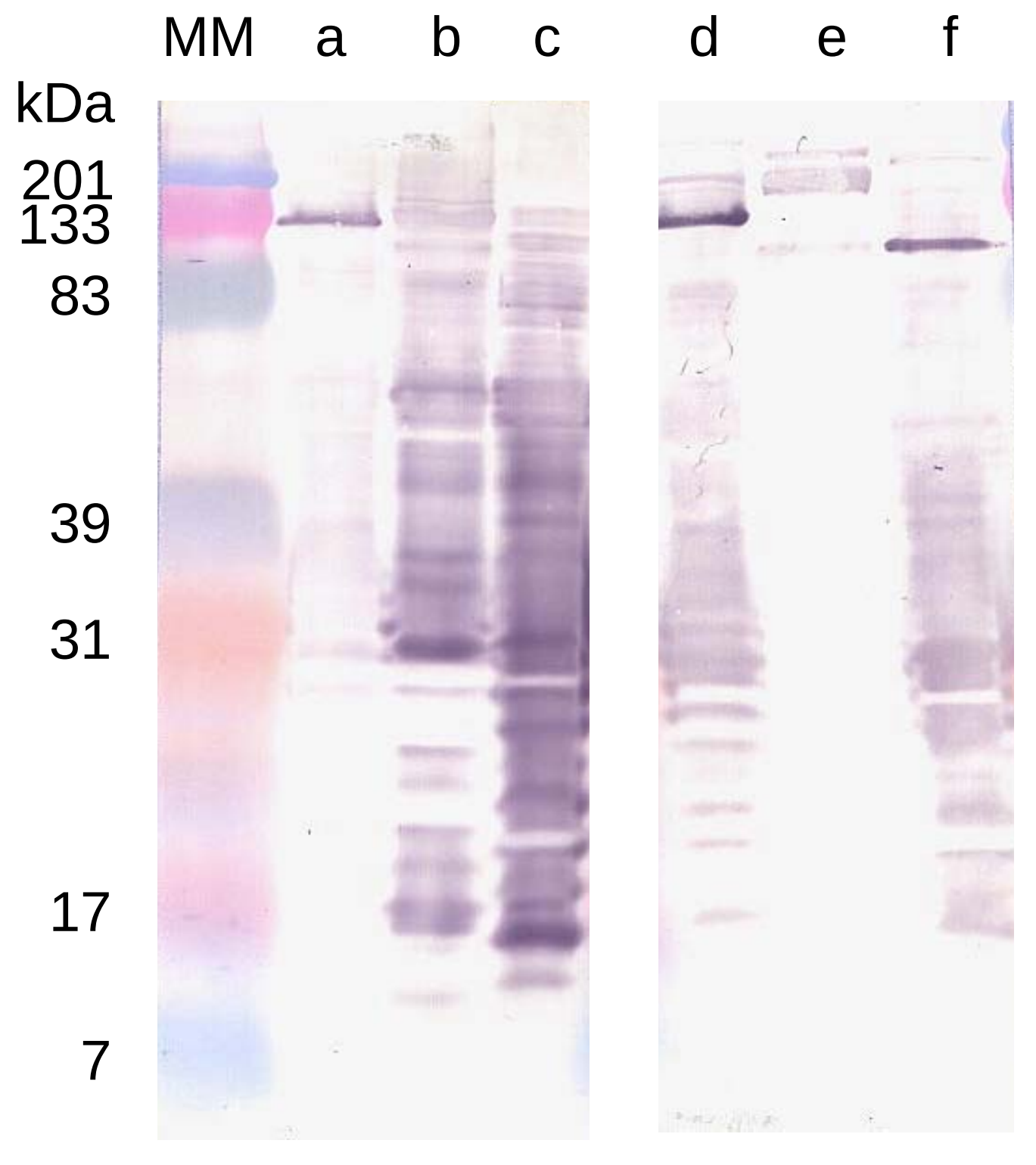

MM a b c d e f
kDa
201
133
83
39
31
17
7
