# Supplementary material for: The Genome Sequence of Rickettsia felis Identifies the First Putative Conjugative Plasmid in an Obligate Intracellular Parasite
Source: PLoS Biol. 2005 Jul 5;3(8):e248. doi: 10.1371/journal.pbio.0030248 (PMC1166351; doi:10.1371/journal.pbio.0030248)
Supplement: Supplementary file 6 [file pbio.0030248.sg007.pdf]

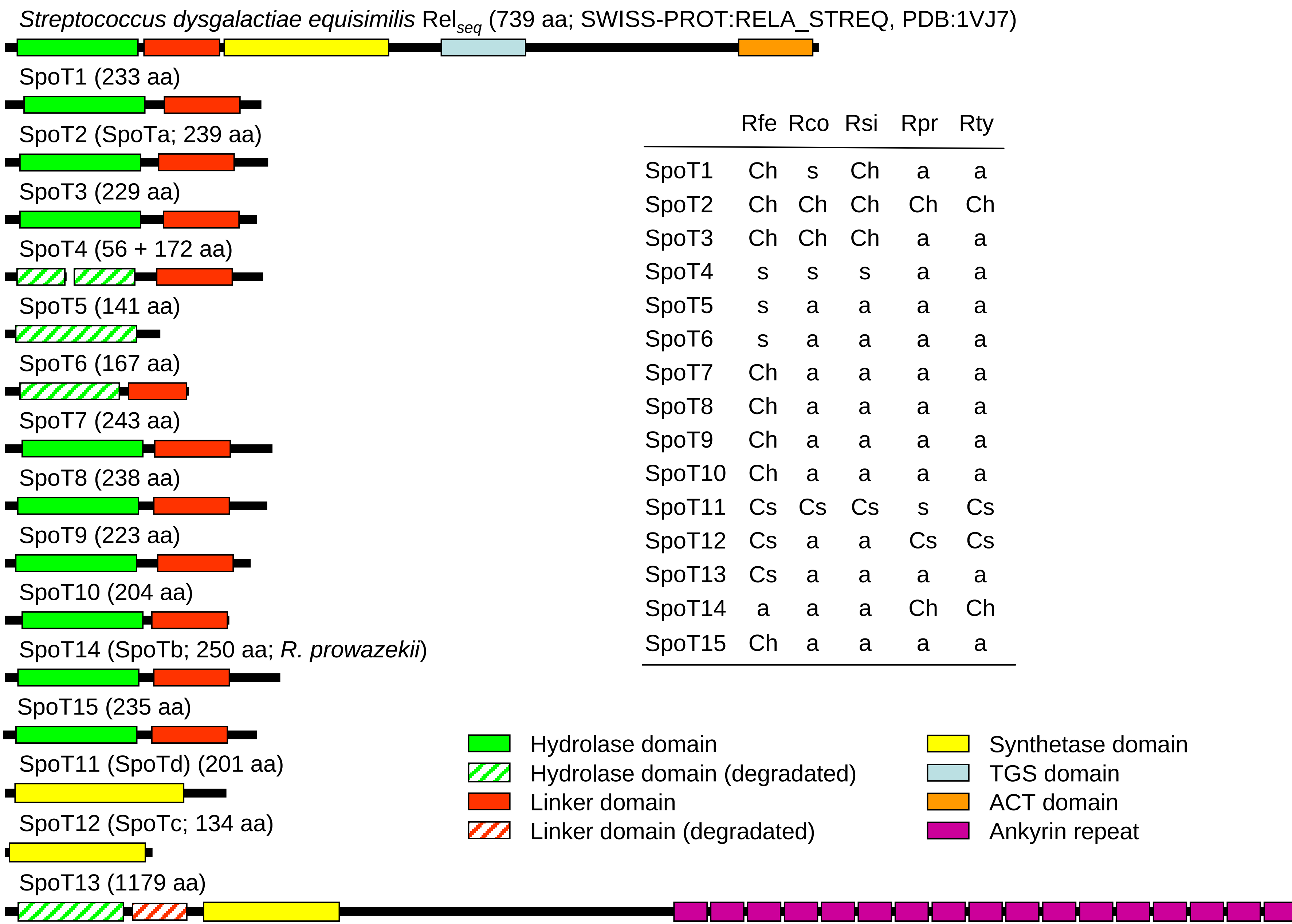

	Rfe	Rco	Rsi	Rpr	Rty
SpoT1	Ch	s	Ch	a	a
SpoT2	Ch	Ch	Ch	Ch	Ch
SpoT3	Ch	Ch	Ch	a	a
SpoT4	s	s	s	a	a
SpoT5	s	a	a	a	a
SpoT6	s	a	a	a	a
SpoT7	Ch	a	a	a	a
SpoT8	Ch	a	a	a	a
SpoT9	Ch	a	a	a	a
SpoT10	Ch	a	a	a	a
SpoT11	Cs	Cs	Cs	s	Cs
SpoT12	Cs	a	a	Cs	Cs
SpoT13	Cs	a	a	a	a
SpoT14	a	a	a	Ch	Ch
SpoT15	Ch	a	a	a	a
