# Supplementary material for: The Genome Sequence of Rickettsia felis Identifies the First Putative Conjugative Plasmid in an Obligate Intracellular Parasite
Source: PLoS Biol. 2005 Jul 5;3(8):e248. doi: 10.1371/journal.pbio.0030248 (PMC1166351; doi:10.1371/journal.pbio.0030248)
Supplement: Supplementary file 8 [file pbio.0030248.st001.doc]

Table S1. Distribution of *sca* genes among *Rickettsia* genomes.

| ***sca* gene** | **Size (aa)*** | **R. felis** | **R. conorii** | **R. sibirica** | ***R. prowazekii*** | **R. typhi** |
| --- | --- | --- | --- | --- | --- | --- |
| *sca*0 (*omp*A) | 1,190 | S (4) | C | C | A | A |
| *sca*1 | 1,703 | C | C | C | S (3) | C |
| *sca*2 | 1,604 | C | C | C | A | A |
| *sca*3 | 3,122 | C | S (1) | S (1) | C | C |
| *sca*4 (gene D) | 1,039 | C | C | C | C¶ | C |
| *sca*5 (*omp*B) | 1,654 | C | C | C | C | C |
| *sca*6 | 1,777 | A | A | A | S (4) | C |
| *sca*7 | 3,180 | S (6) | A | A | A | A |
| *sca*8 | 1,502 | C | S (4) | S (3) | A | A |
| *sca*9 | 557 | C | S (1) | S (1) | A | A |
| *sca*10 | 1,011 | S (3) | S (1) | S (2) | A | A |
| *sca*11 | 333 | C | A | A | A | A |
| *sca*12 | 476 | C | A | A | A | A |
| *sca*13 | 2,199 | C | S (7) | S (9) | A | A |

C = Complete gene; S = Split or fragmented gene; A = Absent. (For split or fragmented genes, the number of ORFs is indicated in parentheses)

* Gene sizes are those of *R. felis*, except for *sca*6 for which we indicated that of *R. typhi sca*6.

¶ According to our recent re-sequencing (GenBank: AY964638)
