# Supplementary material for: The Genome Sequence of Rickettsia felis Identifies the First Putative Conjugative Plasmid in an Obligate Intracellular Parasite
Source: PLoS Biol. 2005 Jul 5;3(8):e248. doi: 10.1371/journal.pbio.0030248 (PMC1166351; doi:10.1371/journal.pbio.0030248)
Supplement: Supplementary file 9 [file pbio.0030248.st002.doc]

Table S2. Comparison of different features of bacteria infecting fleas with their close relatives.

|  | *Rickettsia typhi* | *Rickettsia prowazekii* | *Rickettsia felis* | *Rickettsia conorii* | *Wolbachia pipientis* | *Ehrlichia ruminantium* | Anaplasma marginale | Bartonella henselae | Bartonella quintana | Yersinia pestis | Yersinia pseudotuberculosis |
| --- | --- | --- | --- | --- | --- | --- | --- | --- | --- | --- | --- |
| Arthropod host | Flea | Louse | Flea | Tick | Arthropods (including flea) | Tick | Tick | Flea | Louse | Flea | (Soil, water) |
| Genome size (bp) | 1,111,469 | 1,111,523 | 1,587,240 | 1,268,755 | 1,267,782 | 1,516,355 | 1,197,687 | 1,931,047 | 1,581,384 | 4,829,855 | 4,840,899 |
| Coding content (%) | 73.6 | 76.2 | 83.6 | 81.5 | 85.4 | 62.0 | 86.0 | 72.3 | 72.7 | 83.0 ^†^ | 83.6 ^†^ |
| Plasmid | - | - | + | - | - | - | - | - | - | + | + |
| Repeats (%) | 0.30 | 0.30 | 4.3 | 0.65 | 9.9 | 4.7 | 5.7 | 8.2 | 1.1 | 6.0 | 3.6 |
| Transposases | 0 | 0 | 82 | 0 | 20 | 0 | 0 | 0 | 0 | 220 | 59 |
| Autotransporter domains | 4 | 4 | 12 | 6 | 0 | 0 | 2 | 10 | 5 | 10 | 13 |
| Ankyrin repeats | 1 | 3 | 22 | 2 | 21 | 4 | 3 | 0 | 0 | 1 | 1 |
| Tetratricopeptide repeats | 0 | 0 | 11 | 1 | 2 | 1 | 2 | 3 | 4 | 6 | 6 |
| Integrases | 2 | 2 | 9 | 2 | 3 | 2 | 2 | 4 | 2 | 74 | 28 |

^†^ Coding content for the chromosomes.
