# Supplementary material for: The Genome Sequence of Rickettsia felis Identifies the First Putative Conjugative Plasmid in an Obligate Intracellular Parasite
Source: PLoS Biol. 2005 Jul 5;3(8):e248. doi: 10.1371/journal.pbio.0030248 (PMC1166351; doi:10.1371/journal.pbio.0030248)
Supplement: Supplementary file 10 [file pbio.0030248.st003.doc]

**Table S3. Nucleotide sequences of the primers used in the present study.**

PCR targeting *R. felis* plasmids

traD_pRF37F1	AAAAAGCAGTAGCCTTTGATCG

traD_pRF37R1	AGCTGCTGACCTTTACTTTTCC

pRFa	CAAGCTTTTGTACTGCCTCTAT

pRFb	AGTGCATATAGCTACCACACTATCT

pRFc	ACATTCCGTAAAGAATATGAGC

pRFd	GCTTATGTTCGCCTTTAGTATTTA

pRFe	TAGCATTCATGGCAAACTCT

pRFf	AGTACTTTACCGGGTCTTGC

pRFg	TTCACGTGGTAGTGAAAATTCT

pRFh	ATACCTCATATTGGGTTGAGATAC

pRFi	AGGTATATTATCAATGCCATCTGT

RT-PCR with primers targeting *sca* genes family

RF0022F1	AACACCGGTGACTCCGTATC

RF0022R1	CCCAGCACTGCTCAACAATA

RF0067F1	AAGCAATCGCAGAGCAAGAT

RF0067R1	GCTAATCCACACGCCTCTGT

RF0693F1	TATCGGAGCCGGAGCTAGTA

RF0693R1	GCTGCTCCATTTGCTCCTAC

RF0725F1	TTTAAAATTTGCCGGAGACG

RF0725R1	TTGGTTGTGGTGTTTCCTGA

RF0205F1	GTCACACTTAGCGGCGGTAT

RF0205R1	GTCGAAATCTGTTCCGGCTA

RF1290F1	GTGCAGGTTCTAACGGTGGT

RF1290R1	TCCGCTACCTGAATTTCCAC

RF0473F1	TCCACCATTTGTTGACGGTA

RF0473R1	ACAACACCGGACATTGAACC

RF0474F1	GGAGAGGTTACCGGTGGAGT

RF0474R1	TCCTGTTAGCCCTCCATTTG

RF0475F1	GGAATGTAACCGGAGCGATA

RF0475R1	CCCCGTTAATCCCGTTATTT

RF0476F1	AGTGAAGCTTATGGCCGATG

RF0476R1	AGCATAAGCCGCACCTTCTA

RF0477F1	AGCGGCAGGTGGAGTTACTA

RF0477R1	GTTACCTTGCCCGTTATTCG

RF0478F1	TTGGAACTCGTTATGCTACGTTT

RF0478R1	CCGTGCATCAATATTCGGTA

RF1068F1	CACTCAAATGAGCCGCAATA

RF1068R1	GTTGTCAGAACCGACGTCAA

RF1291F1	ACAACGTTCCTGCGGATACT

RF1291R1	GCCCTGTTGCCCAACTAATA

RF0046F1	GGGGAGATAGGCCTCAAGAT

RF0046R1	TGCAATTCCTCCCATTTCTC

RF0047F1	CGGAGTATCAGCAACGTCAA

RF0047R1	CCGCATGTTTTTCAGGATTT

RF0048F1	TGTTGCAGGTAAATCCGGTA

RF0048R1	TTTCGGCGTGCTACTACTTG

RF0319F1	AACCGTTTGGGTACTGTTGC

RF0319R1	TTTTTGGCTGCTACGCTTAAA

RF1425F1	AAGGCAGGAAAGCAAGATCA

RF1425R1	CACCGCTTAAGGCTTGGTAA

RT-PCR with primers targeting *spoT* genes family

RF0187F1	ATGCTTGCGGAGTTTGTAGC

RF0187R1	TTCCTCGGCACTGATTTTTC

RF0188F1	GCGTTTACGCTAAAAAGCTGA

RF0188R1	CATTTGCAAGCCGTGATATTT

RF0204F1	ACCAAAAAGCAACGGCTATC

RF0204R1	TTTCACCATTTGACGTGCTT

RF0305F1	TCTCAAATGCGTCAATCAGG

RF0305R1	TGACAGCTACCTCTGCTCCA

RF0380F1	AATTGCCAATCAGGTTGAAGA

RF0380R1	TGTTTTCCATCCTTTCTCAGG

RF0381F1	TGTGCTTTTCCGGCAACTAT

RF0381R1	GTTGGTGAGGGTGGCTTAAA

RF0384F1	TCAGATGCGTCAATCAGGAG

RF0384R1	TTTCCAACCAAATTCTTCAGC

RF0410F1	AACAGGTAGGATCAAACATCCAA

RF0410R1	TGATAGCCGTTTGGCTTAGG

RF0438F1	ATTATTCGCACCCGATTGAG

RF0438R1	TGACTCGCGTTAATCCCTCT

RF0440F1	GCTCTTGGAAAGAAAAGTTTGAA

RF0440R1	TAAGCCACCTCAATCGGATG

RF0450F1	CGTCAATCAGGCGATCCTTA

RF0450R1	AAACCCTCTACGTGCTTTGC

RF0508F1	AGAAGCAAAATCGCCTGAAA

RF0508R1	TTTGAGAGAATGGAAAAGAATCG

RF0884F1	CAGGCGATCCGTATTATTCC

RF0884R1	AAACCCTCTACGTGGTTTGC

RF0953F1	TCTCAAATGCGTCAATCAGG

RF0953R1	TTTGCTACTTCCGATCCAAAA

RF0968F1	TCTCATCCTCTTGAAGTTGCAT

RF0968R1	GGGATTGTTTGCCAGAGGTA
